# Ray Tracing versus Thin-Lens Formulas for IOL Power Calculation Using Swept-Source Optical Coherence Tomography Biometry

**DOI:** 10.18502/jovr.v17i2.10788

**Published:** 2022-04-29

**Authors:** Reza Ghaffari, Parisa Abdi, Alireza Moghaddasi, Somayeh Heidarzadeh, Hossein Ghahvhechian, Maryam Kasiri

**Affiliations:** ^1^Eye Research Center, Department of Cornea, Farabi Eye Hospital, Tehran University of Medical Sciences, Tehran, Iran; ^2^Stein Eye Institute and Department of Ophthalmology, David Geffen School of Medicine at the University of California, Los Angeles (UCLA), Los Angeles, California, USA; ^3^Eye Research Center, The Five Senses Health Institute, Rassoul Akram Hospital, Iran University of Medical Sciences, Tehran, Iran

**Keywords:** Biometry, Intraocular, Lenses, Optical Phenomena, Phacoemulsification

## Abstract

**Purpose:**

To evaluate the ray tracing method's accuracy employing Okulix ray tracing software and thin-lens formulas to calculate intraocular lens (IOL) power using a swept-source optical coherence tomography (SS-OCT) biometer (OA2000).

**Methods:**

A total of 188 eyes from 180 patients were included in this study. An OA-2000 optical biometer was used to collect biometric data. The predicted postoperative refraction based on thin-lens formulas including SRK/T, Hoffer Q, Holladay 1, and Haigis formulas and the ray tracing method utilizing the OKULIX software was determined for each patient. To compare the accuracy of approaches, the prediction error and the absolute prediction error were determined.

**Results:**

The mean axial length (AL) was 23.66 mm (range: 19–35). In subgroup analysis based on AL, in all ranges of ALs the ray tracing method had the lowest mean absolute error (0.56), the lowest standard deviation (SD; 0.55), and the greatest proportion of patients within 1 diopter of predicted refraction (87.43%) and the lowest absolute prediction error compared to the other formulas (except to SRK/T) in the AL range between 22 and 24 mm (all *P* < 0.05). In addition, the OKULIX and Haigis formulas had the least variance (variability) in the prediction error in different ranges of AL.

**Conclusion:**

The ray tracing method had the lowest mean absolute error, the lowest standard deviation, and the greatest proportion of patients within 1 diopter of predicted refraction. So, the OKULIX software in combination with SS-OCT biometry (OA2000) performed on par with the third-generation and Haigis formulas, notwithstanding the potential for increased accuracy in the normal range and more consistent results in different ranges of AL.

##  INTRODUCTION

Accurate intraocular lenses (IOL) power calculation and precision refractive outcomes following cataract surgery are now seen as a critical component in determining the success of refractive surgery. In recent years, there have been advancements in this field with the introduction of newer devices for ocular biometry and IOL power calculation formulas. However, choosing the best method for IOL power calculation is still a challenging issue.^[1,2]^

Since 1999, with the introduction of IOL Master, optical biometry has established itself as the standard for axial length (AL) measurement. Since 2009, newer devices such as the AL-Scan (Nidek Co, Aichi, Japan), Lenstar (Haag-Streit, Switzerland), Aladdin (Topcon EU, Tokyo, Japan), and IOL Master 700 (Carl Zeiss Meditec AG, Jena, Germany) have been launched and made accessible. Each of these biometers are made up of four closely related technologies: optical low-coherence interferometry (OLCI), optical low-coherence reflectometry (OLCR), partial coherence interferometry (PCI), and swept-source optical coherence tomography (SS-OCT).^[3,4,5,6]^

The OA-2000 (Tomey, Nagoya, Japan) has lately superseded the OA-1000 model. The OA-2000, which was just released, employs SS-OCT with a laser wavelength of 1060 nm. The AL, anterior chamber depth (ACD), crystalline lens thickness (LT), central corneal thickness (CCT), corneal diameter (CD), pupil size, and keratometry (K) could all be examined with this equipment. ${}^{\left[7,8\right]}$ A firm agreement has also been reported between the OA-2000 and the reference IOL Master 500 biometer for almost all biometry measurements.^[9]^

Concerning the IOL power calculation formulas most commonly used in clinical practice, the thin-lens formulas, including the third-generation theoretical and fourth-generation formulas like Haigis, are currently widely used by cataract surgeons. The Gullstrand eye model is the main structure of these formulas. In this mode, it is assumed that the cornea is a thin optical lens with a refractive index of 1.3375 (or 1.3315 in the Haigis formula) and a constant ratio of the anterior/posterior curvature, along with Gaussian optics assumptions which only apply to paraxial rays, are the basis of all of these formulas.^[2]^

As an alternative approach, ray-tracing technology has been applied for IOL power calculation with promising results in both nonoperated eyes, especially in high myopic and hyperopic patients, and post-refractive surgery eyes. ${}^{\left[10,11\right]}$ The ray-tracing method offers the potential to increase IOL power calculation accuracy by considering the geometric and optical properties of different interfaces like the cornea and the IOL, taking into account the effect of aberrations like spherical aberrations, and performing analyses of single rays limited only by the pupillary zone. ${}^{\left[12,13\right]}$ As a result, in order to design lenses and optical systems, the ray tracing method has now become the standard technique.^[14]^

In this investigation, the OKULIX ray-tracing program was used to assess the accuracy of IOL power calculation, which combines corneal topography data for IOL power calculation, in comparison with the second- and third-generation formulas and the fourth-generation Haigis formula using an SS-OCT biometer (OA- 2000).

##  METHODS

This prospective study was conducted between May 2015 and May 2016. The Institutional Review Board of our Hospital approved the study protocol, and the study was conducted by the tenets of the Declaration of Helsinki. Written informed consent was obtained from all participants.

### Study Population

This study included 188 eyes of 180 patients who had visually significant cataracts. Patients with a history of previous intraocular or refractive surgery, corneal grafts, corneal scars, keratoconus, edema, pseudoexfoliation syndrome, glaucoma, and posterior segment disease (e.g., macular hole, neovascular age-dependent maculopathy, macular edema, or geographic atrophy) were excluded from the study. Patients with intraoperative complications like decentered capsulorhexis, tear in the anterior or posterior capsule, vitreous loss, or those with a postoperative best-corrected visual acuity (BCVA) < 20/40 were excluded as well.

### Preoperative Measurements

Complete ophthalmic examinations, including visual acuity testing, tonometry, and fundus examination, were performed before surgery.

Biometric data including AL, ACD, CCT, and LT were collected using the OA 2000 optical biometer (Nagoya, Japan, software V.1.0R) in the immersion mode. A swept-source laser with a wavelength of 1060 was used to measure the optical distance between ocular surfaces. The device can analyze corneal curvature in nine rings, each with 256 points in a 5.5 mm zone using placido-based topography of the anterior corneal surface. To experience the best correlation with the IOL Master, keratometry values in the 2.5 mm optical zone were utilized for the IOL power calculation for the thin-lens formulae. The IOL power for each patient was chosen in accordance with the intended refraction of 0.00 D.

The IOL power and the predicted postoperative refraction based on thin-lens formulas, third-generation formulas (SRK/T, Hoffer Q, and Holladay 1), and the fourth-generation Haigis formula were determined by the device software for each patient. The optimized IOL constants for each IOL and formula provided by the User Group for Laser Interference Biometry (Available at http://www.augenklinik.uniwuerzburg.de/ulib/c1.htm, accessed April 12, 2017) were used for calculations.

### Ray-tracing Method

OKULIX ray-tracing program (Tedics Peric & Jöher GbR, Dortmund, Germany) was used to assess the IOL power and also to forecast postoperative refraction while relying upon the implanted IOL type used for the ray-tracing method.

OKULIX is a software that calculates the IOL power using corneal topography and has been used extensively in prior cases.^[2]^ The program is capable of modeling the monochromatic optical capacities of the pseudophakic human eye.

Unlike Gaussian optics-based formulas, which are only applicable for paraxial rays, OKULIX performs an analysis of single rays limited only by the pupillary zone, taking into account the effect of spherical aberration. Ray tracing covers the region between the fovea and the cornea. The software also considers the effect of oblique incidence of light rays (Stiles-Crawford effect). Light rays undergo refractions on different interfaces (vitreous, lens, aqueous humor, cornea), and the refractive index changes at each interface. The Okulix database contains labeled IOL data, such as anterior and posterior vertex radii, central thickness, refractive index, and asphericity of both surfaces for aspheric IOLs.

The pupil size in the ray-tracing software was set at 2.5 mm at iris plane. All calculations of the OKULIX were set for the optimal focus, that was described by the International Organization for Standardization (ISO 11979-2) as the ray that contacts the pupil plane at a determined distance ( $d=0.5\times \sqrt{2}\times pupildiameter$ ) for each meridian. The position of the postoperative IOL was determined using an algorithm based on ACD and crystalline LT.^[13]^

### Surgical Technique

All patients underwent phacoemulsification performing a 2.8 mm temporal approach with astigmatically neutral or near-neutral posterior limbal incisions.^[16]^ Aiming at a 5-mm capsulorhexis size, the standard phacoemulsification technique was used to remove the cataract. The following spherical monofocal IOLs, C-flex 570C (Rayner Intraocular Lenses Ltd, East Sussex, UK), Rayner Superflex 620H, and Acry-Sof SA60AT (Alcon Alcon Laboratories Inc, Ft Worth, Texas), and an aspheric IOL, Rayner Superflex Aspheric 920H (Rayner) were selected, assigned, and implanted in the capsular bag of the respective patients. IOL power selection was based on the AL. The SRK-T was used in AL > 26, Holladay 1 for AL between 22 and 26, and the Hoffer-Q in AL < 22. All operations were performed by the same experienced surgeon (RG).

### Outcome Measures

The prediction error (PE) and absolute PE were used to assess accuracy (AE). The difference between the spherical equivalent (SE) of the refraction predicted by the formula for the implanted IOL and the actual postoperative refraction was designated as the PE. Subjective refraction using a 6-m acuity chart was employed to detect manifest refraction one month postoperatively.

**Table 1:** The axial length measurements of patients and the frequency of axial length ranges


Mean	Median	SD	Range	Frequency (%)					
		< 22 mm	22-24 mm	24-26 mm	26-30 mm	> 30 mm	Total
23.66 mm	23.29 mm	± 2.07	19 to 35 mm	22(11.7)	112(59.6)	37(19.7)	13(6.9)	4(2.1)	188(100)
	
	
SD, standard deviation									

**Table 2:** Prediction and absolute prediction error and percentage of patients within the predicted refraction for each formula


**Formula**	**PE**				**AE**				**% Within Predicted Refraction**		
	Mean	SD	Median	Range	Mean	SD	Median	Range	± 0.25D	± 0.50 D	± 1.00 D
**SRK/T**	0.18	1.07	0.18	-4.14 to 4.09	0.75	0.79	0.50	0.01 to 4.14	25.00	50.53	78.19
**Hoffer Q**	0.13	1.06	0.11	-5.11 to 3.97	0.74	0.77	0.48	0.02 to 5.11	20.21	53.19	76.60
**Holladay 1**	0.16	1.02	0.12	-4.72 to 4.03	0.71	0.75	0.48	0.01 to 4.72	24.47	53.72	80.32
**Haigis**	0.24	1.06	0.20	-5.53 to 4.31	0.71	0.82	0.49	0.00 to 5.53	25.00	52.66	80.85
**Okulix**	0.09	0.79	0.01	-1.89 to 1.49	0.56	0.55	0.47	0.01 to 1.89	29.94	55.69	87.43
	
	
PE, prediction error; AE, absolute prediction error; SD, standard deviation											

**Table 3:** Pairwise Comparison of absolute prediction error between Okulix and other formulas


	**Formula**(J)	**Mean Difference(I-J)**	**SE**	***P *** **value**	**95% Confidence Interval for Difference**
	SRK/T	-0.180	0.037	< 0.001	-0.293 to -0.066
**Okulix**(I)	Hoffer Q	-0.168	0.036	< 0.001	-0.279 to -0.057
	Holladay 1	-0.140	0.035	0.002	-0.247 to -0.033
	Haigis	-0.144	0.038	0.003	-0.260 to -0.029
	
	
SE, standard error					

**Figure 1:**
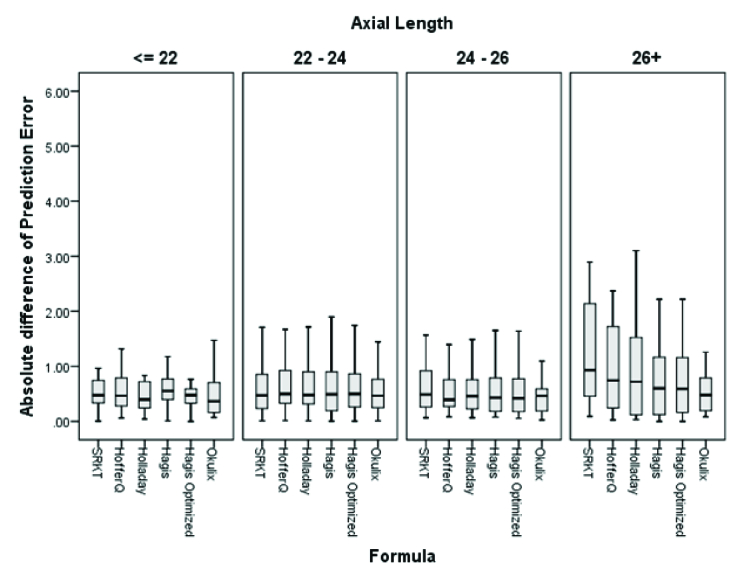
The absolute prediction error of each formula. Comparison of the prediction error of the formulas in subgroup analysis based on the AL using pairwise comparisons with the Bonferroni correction showed that the OKULIX had the lowest MAE compared to other formulas (all *P* < 0.05).

**Figure 2:**
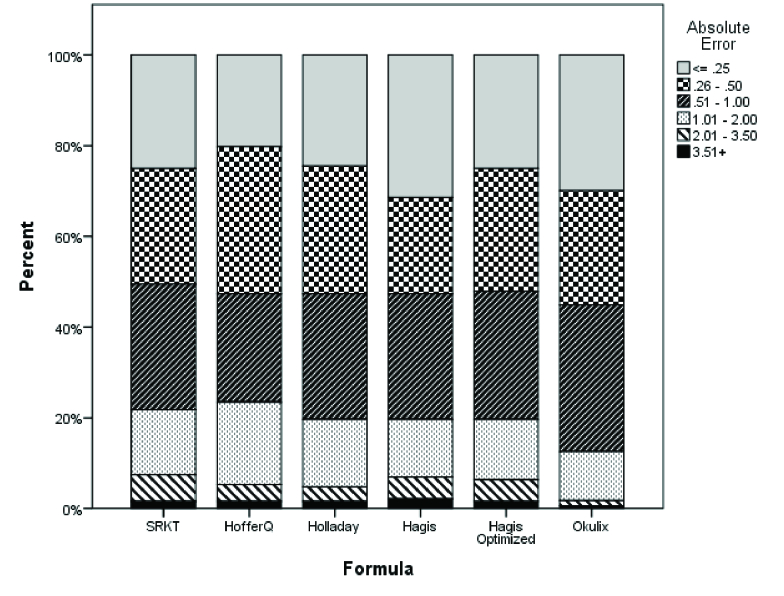
The absolute prediction error of the formulas based on the axial length. There were no statistically significant differences between the other formulas in subgroup analysis based on the axial length.

**Figure 3:**
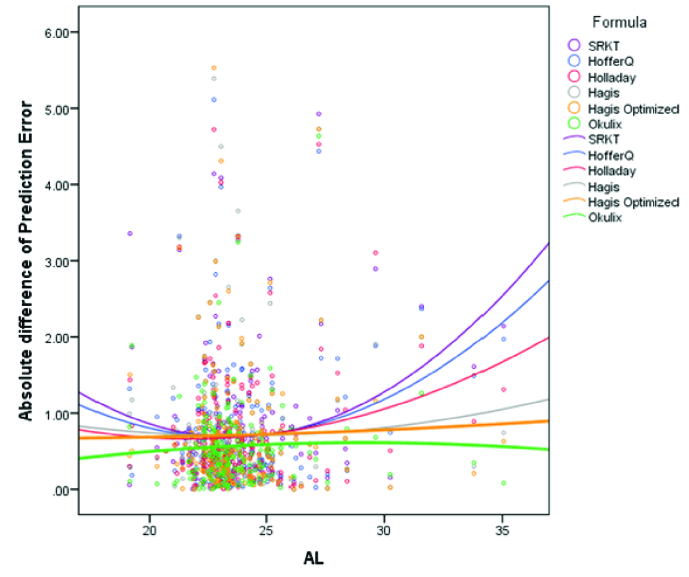
Percentage of eyes within the predicted refraction in each formula. Regarding the percentage of eyes within the predicted refractions of 0.25, 0.5, and 1 D, although the OKULIX had the highest proportion of the eyes in each group, the difference was not significant based on the generalized estimating equation analysis for the percentage of eyes within the 0.25 and 0.5 D. However, there was a significant difference in the percentages of eyes within 1 D of predicted refraction between the OKULIX and all the other formulas (*P* < 0.05).

**Figure 4:**
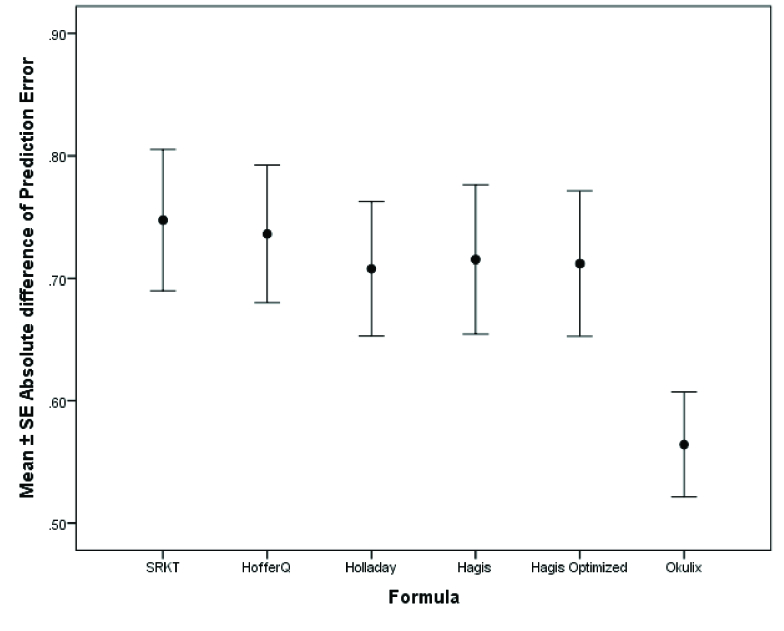
Distribution of absolute prediction errors based on axial length for each formula. The OKULIX and Haigis formulas had the lowest magnitude of change in the absolute prediction error in different ranges of axial length. Most cases of refractive surprise were myopic eyes with ALs > 26 mm.

**Figure 5:**
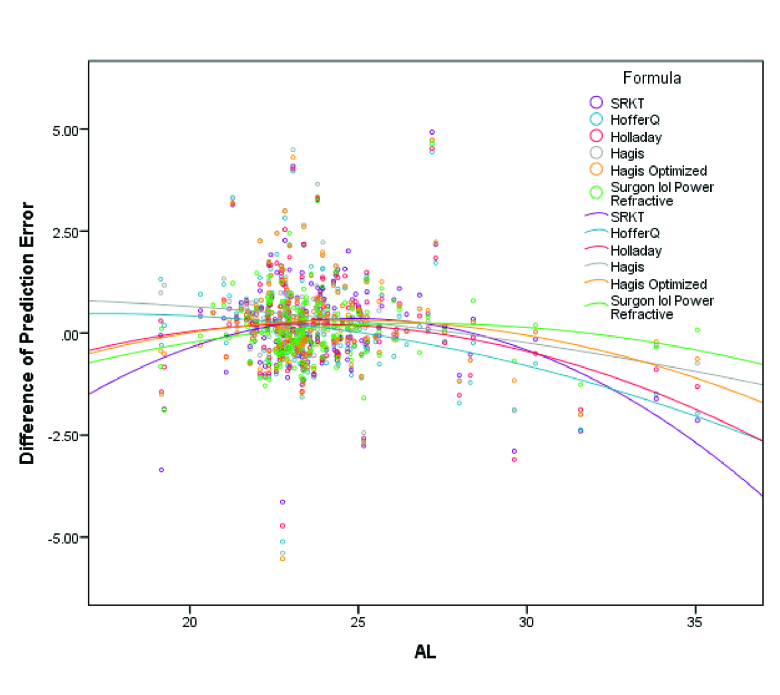
Distribution of absolute prediction errors based on axial length for each formula. The OKULIX and Haigis formulas had the lowest magnitude of change in the prediction error in different ranges of axial length.

### Statistical Analysis

The mean, median, and standard deviation (SD) of PE and absolute PE were calculated to show the distribution of the data. The formulas were compared using linear mixed models and pairwise comparisons with Bonferroni correction after accounting for inter-eye correlation. The heterogeneity of the variances was analyzed by the Leven's Test. Subgroup analysis based on the AL and IOL type was also done by pairwise comparisons with Bonferroni correction. The percentage of eyes within the predicted refractions of 0.25, 0.5, and 1 D for each formula was calculated, and the significance of their differences was analyzed by the generalized estimating equation (GEE) analysis.

##  RESULTS

The study included 188 eyes of 180 patients (100 women; mean age, 62.4 ± 10.4 years). The mean AL was 23.66 ± 2 mm (range: 19–35). Table 1 shows the distribution of the AL in our patients.

The implanted IOL models included the Rayner C-flex 570C (59 eyes), Rayner Superflex 620H (22 eyes), Rayner Superflex Aspheric 920H (70 eyes), and Acry-Sof SA60AT (37 eyes).

The mean power of the implanted IOLs was 18.87 ± 6.12 D (range: –9 to 39) and the mean postoperative manifest refraction was 0.1 ± 0.78 D (range: –4.62 to +2.75). In four patients, the postoperative absolute refractive SE error was more than 2 D.

The mean and SD of the PE and AE of all seven formulas, and the percentage of the patients within the predicted refractions of 0.25, 0.5, and 1 D for each formula have been shown in Table 2.

Comparison of the PE between different formulas showed the significant superiority of the Okulix formula in terms of the lowest mean AE (MAE), the lowest SD, and the narrowest range of PE. The heterogeneity of variances was significantly based on Leven's test (*P* < 0.001).

As shown in Table 3 and Figure 1, pairwise comparisons of the formulas with Bonferroni correction revealed statistically significant lower values in the MAE of the Okulix as compared to other formulas (all *P* < 0.05). There were no significant differences between the SRK/T, Hoffer Q, Holladay 1, Haigis standard, and Haigis optimized formulas.

Comparison of the PE of the formulas in subgroup analysis based on the AL using pairwise comparisons with the Bonferroni correction showed that the Okulix had the lowest MAE as compared to other formulas (Except SRK/T) in the AL range of 22–24 mm (Table 4). There were no statistically significant differences between formulas in the subgroup analysis based on the AL [Figure 2].

Regarding the percentage of eyes within the predicted refractions of 0.25, 0.5, and 1 D, although the Okulix had the highest proportion of the eyes in each group [Table 2 and Figure 3], based on the GEE analysis for the percentage of eyes within the 0.25 and 0.5 D, the difference was not significant. However, there was a significant difference in the percentages of eyes within 1 D of predicted refraction between the Okulix and all the other formulas (*P* < 0.05).

The distribution of the PE and AE error for each formula based on the AL has been shown in Figures 4 and 5, respectively. As shown in these figures, the Okulix and Haigis formulas had the least variability (minimum variance) of change in the PE in different ranges of AL. In subgroup analysis based on the implanted IOL type using the linear mixed model and ad-hoc Bonferroni's test, third-generation formulas did not seem to favor one IOL model over others. There was significant precision calculated for C-flex 570C among other IOLs for the Okulix (*P* < 0.05), however, no statistical difference was found between the Okulix and other formulas in subgroup analysis for other IOLs. The detailed results based on the IOL type have been shown as supplemental data [Table 1].

##  DISCUSSION

Our study results showed that the ray tracing method using the Okulix software together with the OA- 2000 swept-source optical biometer performed comparable other third-generation and Haigis formulas.

The mean AL of our study population was 23.66 mm, which is within the range of the mean AL reported in the normal population. Incidentally, our study included a vast range of ALs (19–35 mm) and patients with short (11.7 % with AL < 22 mm) and long eyes (9% with AL > 26 mm).

In this study, based on a pairwise comparison of the formulas, the Okulix had the lowest absolute error when the analysis included all ALs. However, in subgroup analysis based on AL, the difference in absolute error was statistically significant in the AL 22–24 mm (except to SRK/T). There were no significant differences between the other third-generation and Haigis formulas when the AL was not considered.

The good results of the ray-tracing method may be due to the fact that the ray tracing uses the exact Snell's law as compared to the conventional thin-lens formulas which rely on Gaussian optics assumptions which apply only to the paraxial rays in the optical system. Therefore, it can model the human eye optics more precisely. ${}^{\left[11,17\right]}$ The ray-tracing also incorporates corneal topography data, which means it may be more accurate in calculating corneal power since more wide surface data is entered into the ray-tracing program.^[17]^

Incorporation of the crystalline lens position and thickness as biometric parameters in addition to factors like the AL for prediction of the postoperative pseudophakic ACD could be another reason for improved results of the ray-tracing method in our study. Hoffman et al reported improved results (9% reduction in the MAE) by taking into account the effect of the crystalline lens position and thickness in the algorithm used to predict the postoperative IOL position as compared to the algorithm only using the AL [Appendix1].^[13]^ Some other modern formulas like the Holladay 2 and Olsen also employ the crystalline LT as an important biometric measurement in their calculations for predicting the effective lens position (ELP). This finding is consistent with previous reports about PE of the third-generation formulas. ${}^{\left[18,19\right]}$

Regarding the relationship between the PE and AL changes, the OKULIX and Haigis formulas had the least variance in different ranges of the AL. As shown in Figure 2, in normal and short ALs, and especially in the AL 22–24 mm, there was the least discrepancy between the formulas. As the AL increased, remarkable differences could be observed in each formula's absolute error, so that both the mean absolute error of each formula and also the discrepancy between different formulas increased in ALs > 26 mm. However, the OKULIX had the lowest changes in the absolute error based on the AL. These findings are in agreement with the results of the study by Hoffman.^[20]^ In this study, the Holladay 1 formula in ALs < 24 mm and the Haigis formula in ALs > 24 mm had the lowest error after the OKULIX.

The methods used for prediction of the IOL position in each formula could be a factor explaining differences observed in multiple ALs. Third-generation formulas use different IOL constants, ALs, and keratometry readings for ELP prediction. Using keratometry readings to calculate the corneal height as a basis for this formula is implicated as a source of error in ELP estimation in different combinations of the AL and keratometry, resulting in non-physiologic irregularities in the prediction of the IOL power as described in the SRK/T formula. ${}^{\left[21,22\right]}$ On the other hand, the use of parameters like the AC depth in the Haigis formula [which uses three different constants a0, a1, a2 related to the IOL, AC depth, and AL, respectively] and the AC depth and LT in the OKULIX formula instead of indirect assumptions based on keratometry readings is associated with a higher degree of overall accuracy in the prediction of the IOL position in different ranges of the AL.

Similar to the results reported by Hoffman et al comparing the accuracy of the OKULIX and the third-generation formulas, we observed results that were on par among (and even more accurate results in the AL 22–24 mm) these formulas. However, unlike their results, we did not find any significant superiority of the OKULIX formula in the high myopic and hyperopic eyes, which may be related to the small sample size of these patients in this study population.

According to Figure 3, the number of cases with refractive surprise was also lower in the OKULIX than other formulas. The predicted error was 1 D and lower in 87.5% of the patients based on the OKULIX, while 80.7% of the patients based on the Haigis and 78.2% based on the SRK/T formula were within the PE of 1 D. According to Figure 4, most cases of refractive surprise were myopic eyes with ALs > 26 mm.

Hoffman et al compared the accuracy of the ray tracing between aspheric aberration-correcting and spherical IOLs and reported the particular benefits of the ray tracing method for IOL power calculation for aspheric IOLs.^[20]^ In this study, we did not find any significant differences between aspheric and spherical IOL types, which may be related to the smaller sample size of our study.

The relatively small sample size is the first limitation of this study. We did not use optimization for other third-generation and Haigis formulas for comparisons which may be necessary for the final achievable accuracy with these formulas since the current software for the OKULIX on the OA-2000 device does not allow for optimization. However, because the optimized IOL constants are not usually available to the surgeon, the results of this study are still valid in helping ophthalmologists to choose the appropriate IOL power formula in the clinical setting. In addition, we did not compare our results with some other modern formulas like the Holladay 2, Olsen, and Barret's formulas. Another limitation is the limited follow-up of our patients. IOL position changes due to fibrosis of capsule that could happen in the first three months after surgery may cause refractive changes. However, there are also papers ${}^{\left[20,23--26\right]}$ using refraction at one month as the outcome. It should also be mentioned that using different IOL types could lead to variation in refractive outcomes. However, due to limitations in the power of lenses available, different types of IOL were used in the study.

In summary, the results of this study demonstrated that the ray tracing method using the OKULIX software and the biometric data provided by the new swept-source biometer yielded comparable results with the third-generation and Haigis formulas. Where all patients were included, the OKULIX formula had the lowest mean absolute error and a more constant pattern of performance in different ranges of the AL when compared to other formulas. Based on our results, the ray-tracing method may be an accurate and robust method for calculation of the IOL power in virgin corneas.

##  Financial Support and Sponsorship

None.

##  Conflicts of Interest

The authors declare that there are no conflicts of interest.

##  Appendix 1

The assumed postoperative IOL position is calculated two formulas:^[13]^

1)

A ${}_{a}$ = C ${}_{m}\times$ $\left(\frac{a}{am}\right){}^{0.7}$ + A ${}_{m}$ – C ${}_{m}$ – 0.5 × (d – d ${}_{m}$ )

A ${}_{a}$ = anterior chamber depth with the IOL in

the eye of interest

Cm = distance between the posteriorcornea and center of a 21.00-D IOL in a mean-sized eye (4.6 mm)

a, am = axial length of the eye of interest and a mean-sized eye (23.6 mm)

Am = anterior chamber depth with the IOL model of interest in a mean-sized eye

d, dm = thickness of the IOL of interest and 21.00-D IOL of the same model.

2)

A ${}_{L}$ = A ${}_{L}$ + 0.574 × t ${}_{L}$ – 0.632 – (0.5 × d)

A ${}_{L}$ = anterior chamber depth with the IOL in

the eye of interest

Ap = preoperative anterior chamber depth

t ${}_{L}$ = thickness of the crystalline lens

d = thickness of the IOL
